# Anatomic Variation of Subclavian Artery Visualized on Ultrasound-Guided Supraclavicular Brachial Plexus Block

**DOI:** 10.1155/2014/394920

**Published:** 2014-07-20

**Authors:** Santvana Kohli, Naveen Yadav, Arunima Prasad, Sumantra Sarathi Banerjee

**Affiliations:** Department of Anesthesiology, Jai Prakash Narayan Apex Trauma Centre, All India Institute of Medical Sciences, New Delhi 110029, India

## Abstract

Use of ultrasonography for performance of nerve and plexus blocks has made the process simpler and safer. However, at times, variant anatomy of the visualized structures can lead to failure of blocks or complications such as intravascular injections. This is especially true in case of novice operators. We report a case of a variant branch of subclavian artery, possibly the dorsal scapular artery passing through the brachial plexus nerve bundles in the supraclavicular area. Since this variation in anatomy was visualized in the scout scan prior to the performance of the block, it was possible to avoid any accidental puncture. Hence, a thorough knowledge of the ultrasound anatomy is important in order to identify various aberrations and variations. It is also prudent to perform a preliminary scan, prior to performance of the block to localize the target area and avoid any inadvertent complications.

## 1. Introduction

Supraclavicular brachial plexus block is a commonly performed technique for anesthesia and analgesia of the upper limb. It is often described as the “spinal anesthesia of the arm,” with a profound motor and sensory block [1]. Ultrasound (USG) is being increasingly used in performance of nerve blocks, making the procedures easier and safer. There have been many reports of variations in anatomy of the brachial plexus as visualized on USG. This results in an increase in the failure rate of blocks as well as the incidence of complications, especially with novice USG operators. Variations in the branches of subclavian artery in the supraclavicular fossa may increase the incidence of vascular puncture or intravascular injection of local anesthetic while performing the block. We present a case of a variant branch of the subclavian artery bisecting the brachial plexus in the supraclavicular area.

## 2. Case Report

A 45-year-old adult male, weighing 75 kg, presented with traumatic fracture of left olecranon and was posted for tension band wiring. He was an ASA grade I patient with no other injuries or associated comorbidity and had a good exercise tolerance. All his routine preoperative investigations, including complete blood count, coagulation profile, chest X-ray, and ECG were within acceptable limits. We planned an USG guided supraclavicular brachial plexus block for the patient. The procedure and the use of VAS score were explained to the patient in the preanesthetic visit and consent was taken. He was premedicated with tab. alprazolam 0.5 mg on the night before surgery and tab. ranitidine 150 mg two hours before surgery.

**Figure 1:**
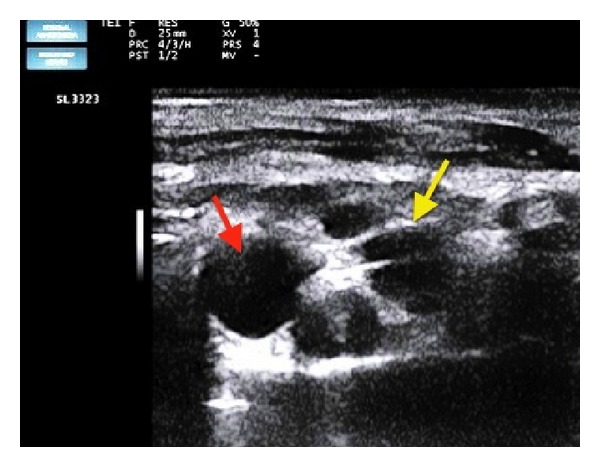
Ultrasound scan of the patient's supraclavicular area showing subclavian artery (red arrow) and brachial plexus divisions (yellow arrow).

**Figure 2:**
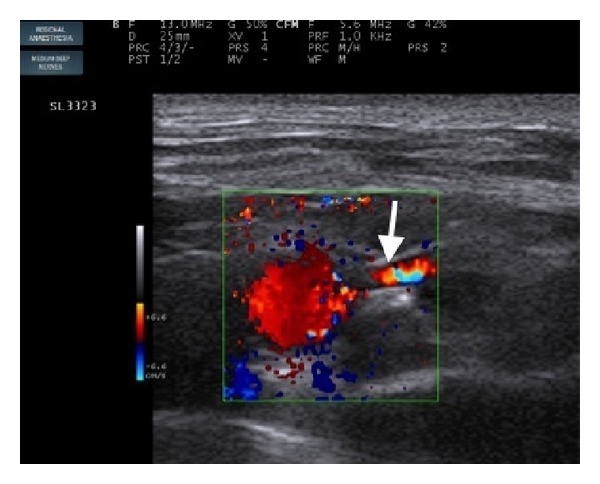
Colour Doppler of subclavian artery showing an aberrant branch (white arrow) in between the divisions of brachial plexus.

On the day of surgery, the patient was taken to the operating room and all monitors (ECG, noninvasive blood pressure and pulse oximetry) were attached. After securing an intravenous (IV) line, ringer lactate infusion was started. Fentanyl 75 *μ*g and midazolam 1.5 mg were given IV. A scout scan of the left supraclavicular area was performed, and the subclavian artery was sought with the divisions of the brachial plexus posterolateral to it (Figure 1). At this point, an aberrant branch of the subclavian artery bisecting the divisions of the brachial plexus was revealed (Figure 2). Block was performed with 25 mL of 0.5% bupivacaine with 75 *μ*g clonidine. Care was taken to avoid puncturing the aberrant branch of the subclavian artery. The onset of block was around 15 minutes, after which the surgery started. The surgery lasted for 90 minutes during which vital parameters of the patient remained stable. VAS score after the surgery was zero and postoperative analgesia (VAS < 4) lasted for 15 hours. The hospital stay was uneventful and the patient was discharged within two days.

## 3. Discussion

Ultrasonography has become an important tool to aid in performance of various nerves and plexus blocks in recent years. It allows anesthesiologists to follow needle trajectory, navigate away from adjacent structures, observe injected solution, and make real-time adjustments that are necessary for effective perineural spread of injectate [2]. A significant benefit of the use of USG in nerve blocks is the identification of vascular structures and other aberrations in the path of the needle. This enables us to avoid these structures and hence decrease the rate of complications.

There have been reports of multiple variant branches of the subclavian artery bisecting the brachial plexus in the neck region and a few in the supraclavicular region. In a cadaveric study carried out by Reiner and Kasser [3], the dorsal scapular artery, found between the trunks of brachial plexus, was found to be originating from subclavian artery in 75% of cadavers. In the remaining 25%, it originated from the transverse cervical artery. In 2005, Weiglein et al. [4] carried out a similar cadaver based study, in which they studied the origin and course of the arteries present in the posterior triangle of the neck. They found that the artery loosely termed as the “transverse cervical artery” was comprised of many different arteries originating from either the subclavian artery or the thyrocervical trunk.

Nambyiah et al. [5] performed a sonographic analysis of the arteries present within the brachial plexus and compared it with cadaveric data. They concluded that vascular structures are present within the brachial plexus in 90% of the subjects, and these can reliably be identified with the help of a USG. They advocated a preprocedural scan while performing a brachial plexus block. Muhly and Orebaugh [6] also studied the sonoanatomy of vasculature in interscalene and supraclavicular areas of 50 patients undergoing shoulder surgeries. They found that an arterial branch, arising from the subclavian artery, passes in between the plexus in the supraclavicular area in a significant proportion of patients.

Many other authors [7–9] have found vascular structures within the substance of the brachial plexus. Many times, these result in an incomplete spread of the local anesthetic agent resulting in what appears as the failure of the blockade. Although a variant branch arising from the subclavian artery was present within the brachial plexus in our patient, there was no obstruction to the spread of injectate.

Hence, it is prudent for all anesthesiologists performing ultrasound-guided blocks to carefully evaluate the sonoanatomy of visualized structures. There is a significant incidence of anatomical variation, which may lead to failure of spread of local anesthetic or may increase the chances of complications, especially, intravascular injection of drug.

## References

[1] Sites BD, Antonakkis JG (2009). Ultrasound guidance in regional anesthesia: state of the art review through challenging clinical scenarios. *Journal of Local and Regional Anesthesia*.

[2] Sites BD, Chan VW, Neal JM (2009). The American Society of Regional Anesthesia and Pain Medicine and the European Society of Regional Anaesthesia and Pain Therapy joint committee recommendations for education and training in ultrasound-guided regional anesthesia. *Regional Anesthesia and Pain Medicine*.

[3] Reiner A, Kasser R (1996). Relative frequency of a subclavian vs. a transverse cervical origin for the dorsal scapular artery in humans. *The Anatomical Record*.

[4] Weiglein AH, Moriggl B, Schalk C, Künzel KH, Müller U (2005). Arteries in the posterior cervical triangle in man. *Clinical Anatomy*.

[5] Nambyiah P, Umbarje K, Amir R, Parikh M, Oosthuysen SAV (2011). Sonographic assessment of arterial frequency and distribution within the brachial plexus: a comparison with the cadaveric record. *Anaesthesia*.

[6] Muhly WT, Orebaugh SL (2011). Sonoanatomy of the vasculature at the supraclavicular and interscalene regions relevant for brachial plexus block. *Acta Anaesthesiologica Scandinavica*.

[7] Abrahams MS, Panzer O, Atchabahian A, Horn J, Brown AR (2008). Case report: limitation of local anesthetic spread during ultrasound-guided interscalene block. Description of an anatomic variant with clinical correlation. *Regional Anesthesia and Pain Medicine*.

[8] Manickam BP, Oosthuysen SAV, Parikh MK (2009). Supraclavicular brachial plexus blockvvariant relation of brachial plexus to subclavian artery on the first rib. *Regional Anesthesia and Pain Medicine*.

[9] Kinjo S, Frankel A (2012). Failure of supraclavicular block under ultrasound guidance: clinical relevance of anatomical variation of cervical vessels. *Journal of Anesthesia*.

